# Impact of Water Deficit Stress on *Brassica* Crops: Growth and Yield, Physiological and Biochemical Responses

**DOI:** 10.3390/plants14131942

**Published:** 2025-06-24

**Authors:** Vijaya R. Mohan, Mason T. MacDonald, Lord Abbey

**Affiliations:** Department of Plant, Food, and Environmental Sciences, Faculty of Agriculture, Dalhousie University, Bible Hill, NS B2N 5E3, Canada; mason.macdonald@dal.ca (M.T.M.); labbey@dal.ca (L.A.)

**Keywords:** drought, water deficit stress, climate change, *Brassica*, photosynthesis, tolerance, mechanisms

## Abstract

Drought including both meteorological drought and water deficiency stress conditions is a major constraint on global agricultural productivity, particularly affecting *Brassica* species, which are vital oilseed and vegetable crops. As climate change intensifies, understanding plant responses to drought is crucial for improving drought resilience. Drought stress impacts *Brassica* crops at multiple levels, reducing germination rates, impairing physiological functions such as photosynthesis and water-use efficiency, and triggering oxidative stress due to the accumulation of reactive oxygen species. To counteract these effects, *Brassica* plants employ various adaptive mechanisms, including osmotic adjustment, antioxidant defense activation, and hormonal regulation. Recent research has explored molecular and physiological pathways involved in drought tolerance, revealing key physiological changes and biochemical markers that could be targeted for crop improvement. This review summarizes the latest findings on the physiological, biochemical, and molecular responses of *Brassica* crops to drought stress, with an emphasis on adaptive mechanisms and potential drought mitigation strategies. Additionally, future research directions are proposed, focusing on integrating molecular and agronomic approaches to enhance drought resilience in *Brassica* species.

## 1. Introduction

The *Brassica*ceae family comprises 351 genera and 3977 species, and some of them are of great economic importance [1]. Some important species of *Brassicas* include *Brassica oleracea* var. *capitata* (cabbage), *Brassica oleracea* var. *botrytis* (cauliflower), *Brassica oleracea* var. *italica* (broccoli), *Brassica oleracea* var. *gemmifera* (Brussels sprouts), and *Brassica oleracea* var. *acephala* (kale). These vegetables are known for their antioxidant and anticancer properties which are proven to offer many nutritional benefits for humans [2,3,4,5]. Except for Antarctica, vegetables of the *Brassica*ceae family are widely distributed around the world. One fascinating aspect of this plant family is its abundance of secondary metabolites which give them their unique flavors and intriguing bioactive properties [6]. *Brassica* species are more susceptible to drought and salt since they are predominantly grown in arid and semi-arid locations [7].

Plants experience a variety of environmental stresses as they grow and develop in different environments. Stress is defined as any adverse condition or agent that disrupts or hinders a plant’s normal metabolism, growth, or development [8,9]. These stresses cause a wide range of plant responses, including changes in gene expression, cellular metabolism, growth rates, and crop yields [10,11,12]. Plants exposed to certain stressors experience metabolic dysfunctions that result in yield loss [8]. Some effects of stress are temporary, and plants may recover if the stress is minimal. Conversely, extreme stress can speed up senescence, prevent flowering and seed production, and eventually cause plant death [13]. Plant stress is broadly categorized into abiotic and biotic stress. Abiotic stress is caused by environmental factors, such as drought and heat, while biotic stress results from biological agents, such as diseases and insect infestations [8].

Due to climate change, global crop yields could decrease by 3–12% by this mid-century and 11–25% by the end of this century [14]. Adding to this concern, climate change is expected to cause extreme temperatures and more severe, prolonged droughts in certain regions, significantly affecting crop growth and productivity [15]. Climate change is increasing the frequency and intensity of droughts globally, with projections indicating that this pattern will likely become even more severe in the future [16]. The term “drought” refers to meteorological drought, defined as a prolonged period of below-average precipitation resulting in limited water availability in soil and atmosphere [17]. Climate change intensifies drought by altering precipitation patterns and raising temperatures. Higher temperatures accelerate evaporation and increase plant water demand, leading to reduced soil moisture and greater water scarcity [18,19]. By the end of the 21st century, over 40% of global land is projected to face a year-round drought, even under low-emissions scenarios [20,21]. Drought remains the most critical abiotic stress, affecting crop and livestock productivity and impacting around 55 million people annually [20,21]. By 2030, the risk of crop yield failures due to drought is projected to be 4.5 times higher than current levels, escalating to 25 times higher by 2050 [22]. Water accounts for 80–95% of fresh biomass in plants and is essential for various aspects of their growth, development, metabolism, and overall function [23]. When this vital resource becomes limited, plant yield is significantly impacted. Drought can result from reduced rainfall and an increased frequency of dry spells, ultimately leading to water scarcity [24]. It is often accompanied by other harmful conditions such as salinity, heat stress, and pathogen attacks [25,26]. In response to these stresses, plants initiate several physiological and morphological adaptations, including stunted root and shoot growth, reduced transpiration and photosynthetic rates, osmotic adjustments, elevated production of reactive oxygen species (ROS), altered stress signaling pathways, and premature senescence [27]. These adaptive changes can ultimately lead to a decline in both the quality and quantity of crop yield [17].

During water deficiency stress, plants undergo an instant physiological response, such as the quick closure of stomata [28]. While this response helps to conserve water, it also simultaneously reduces the intake of carbon dioxide (CO_2_), leading to a decline in photosynthesis (Pn) and subsequent growth retardation [29]. This reduction in CO_2_ uptake creates an imbalance between the energy produced by electron excitation in the light reactions of photosynthesis and the energy used by the Calvin cycle [30]. As a result, there is an overproduction of reactive oxygen species (ROS), which are byproducts of stress responses in plants. These ROS can serve as signaling molecules by indicating stress at the initial stages, but excessive accumulation leads to oxidative damage [31]. This oxidative damage can affect cellular membranes, proteins, lipids, and other important biochemicals in the plant. Eventually, the ability of the plant to survive under these conditions will be impaired [32]. Recent research demonstrates that water deficiency stress-induced oxidative damage in *Brassica* crops occurs rapidly, with ROS accumulation detectable within 1–2 h, lipid peroxidation (measured by malondialdehyde levels) increasing within 3–6 h, and membrane damage (electrolyte leakage) becoming significant within 6–12 h in sensitive varieties like rapeseed [33]. Antioxidant enzymes like superoxide dismutase (SOD) and catalase (CAT) are upregulated within 2–4 h as a defense response, though prolonged stress leads to irreversible cellular damage and yield losses exceeding 30% within 72 h [33]. This rapid oxidative burst aligns with earlier findings that ROS signaling begins in under 30 min [31], highlighting the acute vulnerability of *Brassica* species to water deficiency stress. This stress can also lead to a decline in enzyme activity, as mentioned above, which regulates plant metabolism, [34] ultimately causing a reduction in crop biomass [35]. Apart from meteorological drought, *Brassica* species can also suffer from physiological drought, where plants fail to uptake available soil water effectively due to adverse soil or root conditions. This condition induces similar physiological disruptions as meteorological drought [36]. Understanding the growth, physiological and biochemical mechanisms underlying drought responses is essential for developing effective strategies to mitigate its effects. This review aims to summarize the growth, physiological, biochemical, and yield responses of important *Brassica* species under drought stress and explore potential mitigation strategies.

## 2. Effects of Drought Stress on Brassica Crops

### 2.1. Effects of Drought Stress on Germination

**Figure 1:**
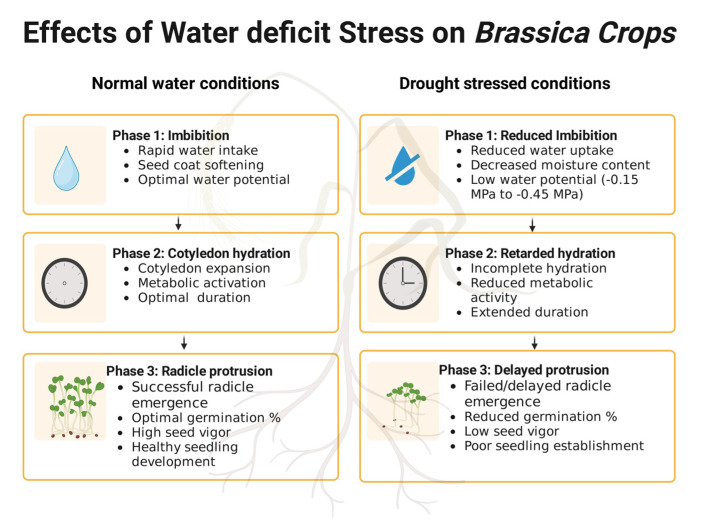
Impact of water deficit stress on sequential germination stages in *Brassica* crops [41,42,43,44,45,46,47,48]. (Created with Biorender.com).

Seed germination starts when a dormant seed absorbs water and concludes with the emergence of a radicle from the seed coat [37]. Seed germination and dormancy are important processes influencing crop production [38]. Seed germination is highly sensitive to environmental factors such as soil moisture, oxygen, and light. The entire growth cycle of *Brassica*s, from germination to harvest, is vulnerable to drought stress [39]. During *Brassica* seed germination, water absorption is the most crucial process and occurs in three distinct phases. The first phase, known as imbibition, involves the seed surface absorbing water. In the second phase, cotyledon hydration takes place, and in the third phase, the radicle emerges, leading to further seedling growth. The third and final phase is the radicle protrusion, as shown in Figure 1 [37].

Disturbances during any stage of seed germination can adversely affect yield [37]. A lower water potential reduces water uptake and moisture content in the first phase, prolongs the second phase, and prevents the seed from progressing to the third phase of imbibition [40]. Insufficient soil moisture negatively impacts seed germination and seedling development [41]. The drought-induced low water potential results in a decreased germination percentage [42]. Studies indicate that limited water availability at the germination stage leads to delayed or reduced seed germination [43]. In addition to germination percentage, seed vigor indices (SVI) provide a more comprehensive assessment of seedling quality, especially under drought stress [44]. Studies have shown that drought stress not only reduces the germination rate but also significantly impairs seedling vigor in *Brassica* species [45,46]. For instance, the germination index and SVI declined progressively with decreasing water potentials such as −0.15 MPa, −0.30 MPa, and −0.45 MPa in rapeseed, indicating a strong negative impact on early seedling vigor [47]. The majority of *Brassica* seeds germinate effectively at a field capacity of 50–75% [48]. Additionally, drought stress often increases soil salinity, further inhibiting seed germination in *Brassicas*. Research on *Brassica* seed germination has shown that as water deficiency stress intensity increases, germination rates decline, which will have a detrimental impact on the overall yields [49].

### 2.2. Effects of Drought Stress on Physiological Characteristics

**Figure 2:**
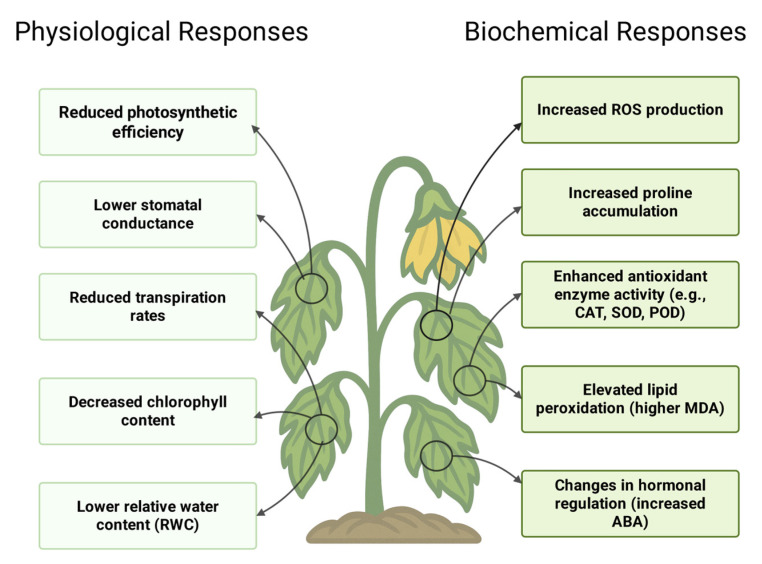
Schematic representation of the physiological and biochemical responses of *Brassica* crops under water deficiency stress conditions [33,55,56,57,58,59,60,61,62,63,64,65,66]. (Created with Biorender.com).

Water is a vital component of cellular structures and plays an important role in all metabolic processes. Photosynthesis is one of several important processes affected by water deficiency stress. Water deficiency stress directly affects the production of photosynthetic products, which serve as the material basis for plant growth and development [29]. It also causes degradation of thylakoid membranes and significant damage to all photosynthetic pigments [50,51]. The net photosynthetic rate, which reflects the productivity per leaf area, is a reliable indicator of a plant’s biological production level [52]. Under water deficiency conditions, both the photosynthetic rate and transpiration rate decline as soil water content decreases [53]. Water is also the primary electron donor in the photosynthetic electron transport chain (ETC); limited water availability disrupts this process, further reducing the plant’s ability to synthesize ATP and NADPH, which are essential for carbon fixation. This reduction in photosynthetic rate initially occurs due to stomatal limitations under mild water deficiency stress, where the closure of stomata reduces CO_2_ uptake, thereby limiting carbon assimilation. However, under severe water deficiency conditions, non-stomatal factors such as impaired chloroplast function, damage to the photosynthetic apparatus (particularly Photosystem II), reduced activity of key enzymes like Rubisco, and increased production of reactive oxygen species (ROS) become the primary causes of the decline in photosynthesis [54]. As a result, the plant cannot carry out its normal functions properly [54]. These physiological and biochemical responses to water deficiency stress in *Brassica* crops are summarized in Figure 2 [33,55,56,57,58,59,60,61,62,63,64,65,66].

Chlorophyll fluorescence analysis has emerged as one of the most powerful and widely used techniques to study the effect of stresses on the photosynthetic process, with the Fv/Fm (maximum photochemical efficiency of PSII) ratio serving as a sensitive indicator of plant photosynthetic performance [67,68]. Under these stresses, *Brassica* crops undergo notable physiological changes primarily related to impaired photosynthetic efficiency [69]. Initial fluorescence (F_0_) generally increases, indicating inhibition at the PSII acceptor side or photo-inhibitory damage, while maximum fluorescence (Fm) declines, signaling reduced PSII efficiency [69]. The Fv/Fm typically decreases under water deficiency conditions especially in *B. oleracea, B. rapa* and *B. napus* [69,70,71], reflecting diminished electron transport capability. Concurrently, photochemical quenching (qP) declines, demonstrating a reduced proportion of open PSII reaction centers, whereas non-photochemical quenching (qN) increases in broccoli and canola plants under water deficiency stress, highlighting greater energy dissipation as heat rather than its utilization in photosynthesis [69,72]. Electron transfer rate (ETR), indicative of photosynthetic electron transport, also tends to decline during water deficiency stress, emphasizing decreased overall photosynthetic performance in *Brassica*s [69]. Additionally, water deficiency stress often reduces chlorophyll content (CCI), although the degree of this response can vary across different *Brassica*s crops [69,73]. It commonly lowers stomatal conductance (gs) and photosynthetic rates (A), accompanied by an increase in internal CO_2_ concentration (Ci), due to reduced assimilation. Studies have shown that water deficiency stress leads to lower stomatal conductance, reduced CO_2_ intake, and decreases in both photosynthesis and transpiration rates in most *Brassica* crops, such as cauliflower and kale, compared to well-watered plants, as shown in Table 1 [61,62,63,74]. Collectively, these physiological responses illustrate significant impacts on broccoli’s photosynthetic system under water deficiency stress.

A drought study conducted on several species of *Brassica*s found that drought-stressed plants had decreased fresh weight, dry weight, and relative water content (RWC) in *B. oleracea* and *B. carinata* [33]. A study found that a 4% reduction in RWC significantly increased stomatal resistance, ranging from 1.89 to 2.94 cm s^−1^ in *Brassica* species [76]. Notably in rapeseed, an 80% decline in RWC resulted in over a 50% reduction in photosynthesis. Additionally, various rapeseed cultivars also exhibited decreases in RWC under water deficit conditions [77,78]. These findings highlight the critical role of RWC in maintaining physiological functions under water deficit conditions. Reduced RWC not only disrupts stomatal regulation but also impairs photosynthetic efficiency, ultimately affecting plant growth and productivity.

### 2.3. Effects of Drought Stress on Oxidative Stress and Antioxidant Defense Systems

Water deficiency and physiological drought conditions lead to oxidative damage in plant cells by triggering the production of ROS, including superoxide radicals, hydrogen peroxide, hydroxyl radicals, and singlet oxygen [31,79]. The accumulation of ROS negatively impacts various physiological and metabolic functions, such as photosynthesis and antioxidant defense [79]. Increased ROS result in lipid peroxidation, chlorophyll degradation, membrane destabilization, and ion leakage, indirectly influencing the overall yield [80,81]. Under normal conditions, cellular ROS levels remain stable due to a balance between their production and scavenging. However, stress factors like drought disrupt this equilibrium, leading to excessive ROS accumulation and oxidative stress [79]. An initial increase in ROS generation, before surpassing the scavenging capacity, can function as a signaling mechanism for defense responses. This signaling role is particularly evident in plant defense against pathogens, where oxidative stress activates protective pathways [82]. Likewise, plants possess internal mechanisms to cope with mild water deficit stress conditions, allowing them to maintain growth and function. However, once the stress exceeds a critical threshold, it leads to adverse effects on growth and yield [53].

A study conducted with *B. oleracea* (cabbage), *B. carinata* (Ethiopian mustard), *B. nigra* (black mustard), *B. napus* (rapeseed), *B. rapa* (field mustard), and *B. juncea* (Indian mustard) found that water deficit conditions led to increased ROS accumulation [33]. Water deficiency stress led to a 1.4-fold increase in malondialdehyde (MDA) level in cabbage, Ethiopian mustard, black mustard, and rapeseed, while hydrogen peroxide (H_2_O_2_) levels were increased 1.2-fold in stressed cabbage and Ethiopian mustard, and amounts of superoxide were increased 1.5-fold in stressed field mustard, cabbage, Ethiopian mustard, black mustard, rapeseed, and Indian mustard [33]. Peroxidase (POD) utilizes various reductants to convert H_2_O_2_ into water in plant cells as a defense mechanism against water deficiency stress [83]. The study found that this stress selectively increased the activities of superoxide dismutase (SOD) and guaiacol peroxidase (POD) while reducing catalase (CAT) activity in ten cultivars of *Brassica napus* L. [84]. Some of the other important biochemical responses are listed in Table 2 and Figure 2.

### 2.4. Effects of Drought Stress on Proline

Proline accumulation is a significant response of plants under water deficiency stress. Proline is a low-molecular compound that accumulates in the cytoplasm of plant cells to enhance their tolerance to the severe impacts of water deficiency stress [91,92]. Proline maintains subcellular structures, including membranes and proteins, and acts as an osmolyte to help with osmotic adjustment [93]. Proline acts as a compatible protein by regulating and triggering various responses, such as scavenging free radicals and maintaining cellular redox balance, enabling plants to withstand abiotic stress [94]. Therefore, proline is mostly recognized as a reliable indicator of environmental stress in plants. Proline accumulation was observed in Chinese cabbage, rapeseed, broccoli, and several other *Brassica* cultivars as a response to water deficiency stress, as shown in Table 3 and Figure 2 [33,55,56]. As a result, the increased proline content in these *Brassica* species contributes to their enhanced tolerance to water deficiency stress, allowing them to survive and maintain growth under challenging conditions. In conclusion, water deficiency stress induces oxidative damage in *Brassica* species, but their ability to accumulate proline, enhance antioxidant enzyme activity, and regulate ROS levels helps improve their tolerance and resilience. These adaptive mechanisms are crucial for maintaining growth and yield under water deficiency stress conditions.

### 2.5. Effects of Drought Stress on Yield Characteristics

**Figure 3:**
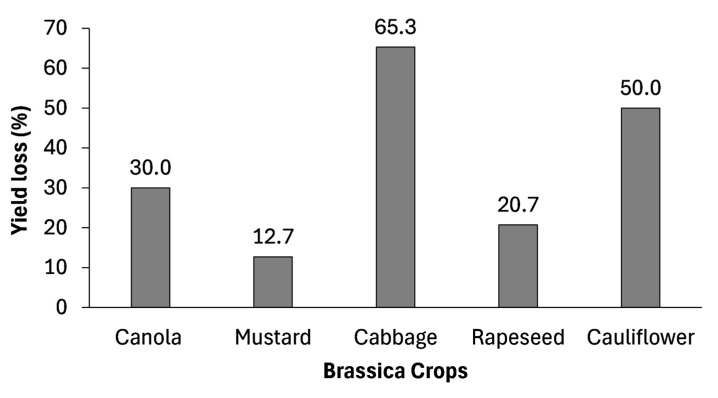
Percentage yield loss in major *Brassica* crops under drought stress conditions. For cauliflower, the yield reduction was estimated by interpolating the reported “40–60% reduction” to midpoint for graphing. This figure was created from data presented by [35,58,86,88,94,95].

Drought stress significantly limits *Brassica* crop production and yield worldwide. For instance, canola suffers an annual yield loss of at least 30% due to drought [35]. Scientific studies have consistently demonstrated that drought stress adversely affects both shoot and root biomass in *Brassica* crops. A study on *Brassica* species found that increasing severity of drought led to a significant reduction in shoot fresh and dry weights [39]. Fresh and dry weights of both shoot and root in cauliflower cultivars decreased considerably under drought stress, with water scarcity drastically reducing curd fresh weight [86]. As illustrated in Figure 3, yield losses across major *Brassica* crops under drought stress conditions can range from moderate to severe, with some reductions reaching up to 65%.

Likewise, total head yield in field-grown cabbage declined from 50.5 t ha^−1^ to 17.5 t ha^−1^ due to drought stress [94]. Similarly, moisture deficit conditions resulted in a 12.7% reduction in mustard yield [58]. The impact of drought stress is particularly severe during the flowering and pod formation stages, the most drought-sensitive phases in rapeseed, leading to yield losses of 30.3% and 20.7%, respectively [88]. Furthermore, limited water availability negatively affects vegetative growth, reducing the number of leaves and branches while slowing overall development, ultimately decreasing yield in cabbage, kale, mustard, and broccoli [62,89,96,97,98,99]. Interestingly, while drought stress significantly increases root length in cauliflower, this adaptation is insufficient to prevent yield loss [95]. These findings highlight the urgent need for developing drought-resistant *Brassica* cultivars and improved water management strategies to mitigate yield losses and improve productivity.

Overall, the drought responses of individual *Brassica* species differ significantly in both the magnitude and type of physiological and biochemical adaptations. As shown in Table 3, field mustard exhibits a markedly higher proline accumulation of up to 435%, compared to more moderate increases of 102% in Chinese cabbage and 74% in canola. In contrast, the lowest increases were observed in kale and white cabbage, at just 36% and 38%, respectively [33,55,56,57,58,59,60]. Similarly, enzymatic antioxidant responses such as guaiacol peroxidase (GPX), superoxide dismutase (SOD), and catalase (CAT) show notable variation among species (Table 2). For example, GPX activity increased in rapeseed, mustard, and cauliflower under drought stress, whereas it decreased in broccoli [58,84,86,87]. Yield-related parameters also display considerable interspecific variability (Figure 3). Cabbage showed a yield reduction of over 65.3% under drought conditions, while mustard demonstrated greater physiological resilience, with only a 12.7% decline in yield under similar conditions [35,58,86,88,94,95]. These differences highlight that drought tolerance mechanisms in *Brassica* crops are highly species- and cultivar-dependent, emphasizing the importance of targeted selection and breeding of drought-resilient genotypes suited to water-limited environments.

## 3. Research Gaps and Future Perspectives

Drought can severely impact crop yields, but there are several strategies to mitigate losses. These strategies can be grouped into agronomic practices, soil and water management, crop selection, and technological interventions [100]. Recent advancements in drought-resistant *Brassica* crops, such as canola and mustard, focus on genetic modifications, selective breeding, and agronomic improvements [101].

### 3.1. Agronomic Practices

Agronomic interventions such as optimized planting dates, row spacing, and mulching can alleviate the effects of drought by improving water retention and plant spacing for better moisture use efficiency [102]. Recent studies emphasize the importance of integrating mulching and irrigation scheduling, but these need validation across diverse agro-ecological zones [53]. However, these techniques are often location-specific, and a major research gap lies in their scalability across agro-ecological zones. There are limited data available on how different mulching materials influence drought resilience in *Brassica*s across varying soil types.

### 3.2. Soil and Water Management

Drip irrigation, partial root-zone drying (PRD), and the application of biochar [96] techniques have shown promise for *Brassica* cultivation, all of which enhance soil moisture retention and optimize plant–water relationships [76,96,103]. Research has demonstrated that PRD can effectively maintain cabbage yields while significantly reducing water consumption [96]. Another study on regulated deficit irrigation in rapeseed further confirmed that strategic water reduction does not necessarily compromise yield outcomes [76]. Despite these encouraging findings, there remains a notable gap in long-term research examining root–soil–water interactions specifically in *Brassica* crops under these irrigation regimes for drought mitigation.

### 3.3. Crop Selection and Breeding

Breeding drought-resilient *Brassica* cultivars has traditionally relied on phenotypic selection, focusing on visible traits such as plant height, biomass, and leaf rolling under water stress. Direct breeding efforts focus on incorporating drought-tolerant traits into elite cultivars. This involves crossing drought-tolerant wild relatives or landraces with high-yielding but drought-sensitive varieties, followed by selection for progeny that combine both traits [104]. This method has generated some success particularly in Indian mustard and rapeseed [105]. However, phenotypic traits are highly influenced by environmental variability, often lacking consistency across growing conditions, which slows the progress of breeding programs. As a result, breeding programs must move toward more rigorous and efficient phenotyping strategies to accelerate the gains [106]. Phenotyping remains a critical link in connecting genotype to phenotype, especially when establishing the genetic basis of climate resilience. The effectiveness of a phenotyping method depends heavily on the breeding objective and the stage of cultivar development [106]. The choice of screening methods and breeding strategies should align with the specific objectives and resources of the breeding program. *Brassica* oilseed crops, particularly Indian mustard, are frequently exposed to unpredictable climatic fluctuations such as drought throughout their growth period. Consequently, developing climate-resilient varieties of oilseed mustard is crucial for maintaining sustainable agricultural productivity and mitigating stresses [107]. In conclusion, enhancing drought tolerance in *Brassica* crops requires a synergistic approach that combines advanced phenotyping, genomic tools, and targeted breeding strategies. By tailoring these methods to specific environmental conditions and breeding objectives, it is possible to develop resilient *Brassica* cultivars capable of maintaining yield stability under drought stress.

### 3.4. Technological Interventions

Technological interventions for drought mitigation in *Brassica* crops include gene editing, seed priming, and the exogenous application of stress-mitigating agents. Among the most transformative advances is the use of CRISPR-Cas9 gene editing to enhance root architecture and water-use efficiency in some of the *Brassica*s to improve their drought resilience [108]. Global agriculture market trends show a rising demand for drought-tolerant *Brassica* varieties, particularly in oilseed production, as climate change drives the need for resilient crops. Seed priming is an effective technique to improve crop resilience to drought [109]. It involves pre-soaking seeds in water to kick-start germination, which can significantly enhance overall yield by promoting early plant development and improving stress tolerance [109]. Priming seeds with selenium and ascorbic acid has shown notable improvements in germination and stress tolerance in radish and broccoli, respectively [85,110,111]. The exogenous application of proline, glutathione, glycine betaines, and salicylic acid has been found to boost drought tolerance in *Brassica napus*, enhancing plant resilience under water stress in cabbage, and using plant growth-promoting rhizobacteria (PGPR), specifically Bacillus megaterium, has significantly increased drought tolerance by promoting root growth, nutrient uptake, and stress-adaptive mechanisms [112]. These methods help mitigate drought stress by improving osmotic balance, strengthening antioxidant defense systems, and fostering beneficial microbial interactions in the rhizosphere, ultimately leading to better crop establishment and yield stability under water-limited conditions [56]. These developments highlight the integration of biotechnology, traditional breeding, and molecular biology to enhance drought resistance in *Brassica*s, ensuring stable yields under fluctuating environmental conditions.

## 4. Conclusions

Drought stress is one of the most significant abiotic factors limiting global crop productivity, particularly affecting *Brassica* species, which are highly vulnerable to water scarcity. This review highlights the diverse impacts of drought on *Brassica* crops, covering effects from germination to physiological, biochemical, and yield responses. Drought stress disrupts essential physiological processes such as photosynthesis, stomatal conductance, and water balance, resulting in reduced growth and productivity. It also triggers biochemical responses, including the overproduction of reactive oxygen species (ROS), oxidative stress, and changes in antioxidant enzyme activity, which further harm plant health and yield. Despite these challenges, *Brassica* species have adaptive mechanisms to reduce drought-induced damage, such as proline accumulation, activation of antioxidant defenses, and hormonal regulation. Understanding these responses is crucial for developing drought-tolerant cultivars through advanced breeding techniques and effective agronomic strategies. Future research should focus on integrating molecular, physiological, and agronomic approaches to enhance drought resilience in *Brassica* crops. Addressing drought stress through sustainable agricultural practices and biotechnological innovations will be key to ensuring food security and sustaining global *Brassica* production in the face of climate change.

## Figures and Tables

**Table 1:** Photosynthetic parameters affected by water limitation in *Brassica* crops: The effects illustrated in the table vary depending on the crop and severity of water deficiency stress.

Photosynthetic Parameter	Decrease (%)	Crops	References
Internal CO_2_ concentration	40–60%	Kale, Cauliflower, Mustard	[61,64,75]
Net CO_2_ assimilation rate	50–70%	Cauliflower, Chinese Cabbage, Cabbage	[62,63,64]
Net photosynthesis	30–80%	Kale, Broccoli, Cauliflower, Mustard	[61,64,65,66,75]
Stomatal conductance	60–90%	Cauliflower, Chinese Cabbage, Cabbage, Kale, Broccoli, Mustard	[61,62,63,64,65,66]
Transpiration rate	50–75%	Chinese Cabbage, Kale, Broccoli, Cauliflower, Mustard	[61,62,64,65,66,75]

**Table 2:** Summarizing the biochemical effects of water deficiency stress on *Brassica*s.

Parameter	Effects	Crops	References
Volatile organic compound (VOC) emissions	Increased (47–275%)	Cabbage, Cauliflower	[63]
Total phenolic content	Increased (50–105%)	Cabbage, Broccoli, Cauliflower	[63,85,86]
Flavonoid content	Increased (85–95%)	Cabbage, Cauliflower, Broccoli	[63,85]
Chlorophyll a, b and carotenoid content	Decreased (13–45%)	Cabbage, Cauliflower, Broccoli, Rapeseed	[57,58,63,85,87,88]
Guaiacol peroxidase (GPX)	Increased (55–150%)	Rapeseed, Mustard, Cauliflower	[58,84,86]
	Decreased (25%)	Broccoli	[87]
Ascorbic acid (AA)	Increased (150–600%)	Broccoli, Chinese cabbage, White cabbage, Kale	[59,85]
Malondialdehyde (MDA)	Increased (92–130%)	Rapeseed, Chinese cabbage, White cabbage, Kale	[59,88]
Catalase (CAT) activity	Increased (11–75%)	Broccoli, Cauliflower, Chinese cabbage, White cabbage, Kale	[59,86,87,89]
Superoxide dismutase (SOD) activity	Increased (14–110%)	Broccoli, Cauliflower, Chinese cabbage, White cabbage, Kale, Rapeseed	[59,84,86,87,89]
Abscisic acid (ABA)	Increased (230–400%)	Chinese cabbage, White cabbage, Kale, Rapeseed	[59,90]
Total cytokinins (CKs)	Increased (50–70%)	Chinese cabbage, White cabbage, Kale	[59]

**Table 3:** Summarizing the percentage increase of proline under water deficiency stress on *Brassica*s [33,55,56,57,58,59,60].

*Brassica*s	Percentage Increase of Proline (%)
Black Mustard	263
Cabbage	50
Canola	74
Chinese Cabbage	102
Ethiopian Mustard	54
Field Mustard	435
Kale	36
Rapeseed	69
Rapeseed	292
White Cabbage	38

## Data Availability

No new data were produced in this research.
